# Bioinspired Green Synthesis of Silver Nanoparticles Using Three Plant Extracts and Their Antibacterial Activity against Rice Bacterial Leaf Blight Pathogen *Xanthomonas oryzae* pv. *oryzae*

**DOI:** 10.3390/plants11212892

**Published:** 2022-10-28

**Authors:** Ye Tian, Jinyan Luo, Hui Wang, Haitham E. M. Zaki, Shanhong Yu, Xiao Wang, Temoor Ahmed, Muhammad Shafiq Shahid, Chengqi Yan, Jianping Chen, Bin Li

**Affiliations:** 1 State Key Laboratory of Rice Biology and Ministry of Agriculture Key Laboratory of Molecular Biology of Crop Pathogens and Insects, Institute of Biotechnology, Zhejiang University, Hangzhou 310058, China; 2 Department of Plant Quarantine, Shanghai Extension and Service Center of Agriculture Technology, Shanghai 201103, China; 3 Horticulture Department, Faculty of Agriculture, Minia University, El-Minia 61517, Egypt; 4 Applied Biotechnology Department, University of Technology and Applied Sciences-Sur, Sur 411, Oman; 5 Taizhou Academy of Agricultural Sciences, Taizhou 317000, China; 6 Ningbo Jiangbei District Agricultural Technology Extension Service Station, Ningbo 315033, China; 7 Department of Plant Sciences, College of Agricultural and Marine Sciences, Sultan Qaboos University, Al-khod 123, Oman; 8 Institute of Biotechnology, Ningbo Academy of Agricultural Sciences, Ningbo 315040, China; 9 State Key Laboratory for Managing Biotic and Chemical Threats to the Quality and Safety of Agro-Products, Key Laboratory of Biotechnology in Plant Protection of Ministry of Agriculture and Zhejiang Province, Institute of Plant Virology, Ningbo University, Ningbo 315211, China

**Keywords:** AgNPs, antibacterial activity, Arctium lappa, Solanum melongena, Taraxacum mongolicum, *Xanthomonas oryzae* pv. *oryzae*

## Abstract

Rice bacterial leaf blight caused by *Xanthomonas oryzae* pv. *oryzae* (Xoo) is responsible for a significant reduction in rice production. Due to the small impact on the environment, biogenic nanomaterials are regarded as a new type of antibacterial agent. In this research, three colloids of silver nanoparticles (AgNPs) were synthesized with different biological materials such as *Arctium lappa* fruit, *Solanum melongena* leaves, and *Taraxacum mongolicum* leaves, and called Al-AgNPs, Sm-AgNPs and Tm-AgNPs, respectively. The appearance of brown colloids and the UV-Visible spectroscopy analysis proved the successful synthesis of the three colloids of AgNPs. Moreover, FTIR and XRD analysis revealed the formation of AgNPs structure. The SEM and TEM analysis indicated that the average diameters of the three synthesized spherical AgNPs were 20.18 nm, 21.00 nm, and 40.08 nm, respectively. The three botanical AgNPs had the strongest bacteriostatic against Xoo strain C2 at 20 μg/mL with the inhibition zone of 16.5 mm, 14.5 mm, and 12.4 mm, while bacterial numbers in a liquid broth (measured by OD_600_) decreased by 72.10%, 68.19%, and 65.60%, respectively. Results showed that the three AgNPs could inhibit biofilm formation and swarming motility of Xoo. The ultrastructural observation showed that Al-AgNPs adhered to the surface of bacteria and broke the bacteria. Overall, the three synthetic AgNPs could be used to inhibit the pathogen Xoo of rice bacterial leaf blight.

## 1. Introduction

Rice bacterial leaf blight caused by *Xanthomonas oryzae* pv. *oryzae* (Xoo) severely damages rice production [1,2,3]. Chemical bactericides have played a vital role in the control of this disease. However, owing to the emergence of bacterial drug resistance and environmental hazards, the use of chemical bactericides is limited [4,5]. Antagonistic bacteria have been used to control this disease, but the inhibitory effect depends on the ecological environments [6,7]. AgNPs have attracted extensive attention recently due to their application in the agricultural field. AgNPs can inhibit plant pathogenic bacteria, fungi, viruses, and nematodes [8,9,10,11]. Research shows that AgNPs inhibit *Klebsiella pneumonia*, *Bacillus subtilis*, *Pseudomonas aeruginosa*, and *Staphylococcus aureus* [12,13,14,15]. In addition, AgNPs can synergistically inhibit bacteria with antibiotics [16].

In the last few years, AgNPs has been synthesized via biological, chemical, and physical methods [17,18,19,20,21]. Among them, biological methods have attracted more attention due to their advantages of being environmentally friendly, simple, fast, stable, and economical [22]. In particular, plant extracts containing alkaloids, polyphenols, polysaccharides, flavonoids, terpenoids, saponins, and tannins serving as reducing agents and stabilizers, which are crucial for the formation of AgNPs [22,23,24,25,26,27]. Indeed, plant-based AgNPs have the characteristics of biocompatibility, environmental protection, and safety. They are also more suitable for large-scale production due to the relatively high reaction rate and simple synthesis conditions [28,29].

Many plant materials have been reported to be useful for synthesizing AgNPs, such as *Capsella bursa-pastoris*, *Emblica officinalis*, *Lysiloma acapulcensisetc*, *Phyla dulcis*, etc. [30,31,32,33,34]. Although biological methods have successfully synthesized many nanoparticles, people are still looking for new nanoparticles with more accurate properties. Interestingly, *A. lappa*, *S. melongena* and *T. mongolicum* are beneficial to human health, and according to reports, they have antioxidant, anti-inflammatory and antibacterial activities [35,36]. *A. lappa*, *S. melongena* and *T. mongolicum* have been used to synthesize various nanoparticles, [37,38,39]. However, the three AgNPs synthesized by *A. lappa*, *S. melongena* and *T. mongolicum* have not been reported to inhibit Xoo.

In this study, we synthesize AgNPs using *A. lappa* fruit, *S. melongena* leaves, and *T. mongolicum* leaves and evaluates their antibacterial mechanisms against rice pathogen Xoo. Overall, the current study develops an economic, efficient, safe, and environmentally friendly nano-formulation to control rice bacterial leaf blight.

## 2. Results and Discussion

### 2.1. Green Synthesis and Characterization of AgNPs

**Figure 1:**
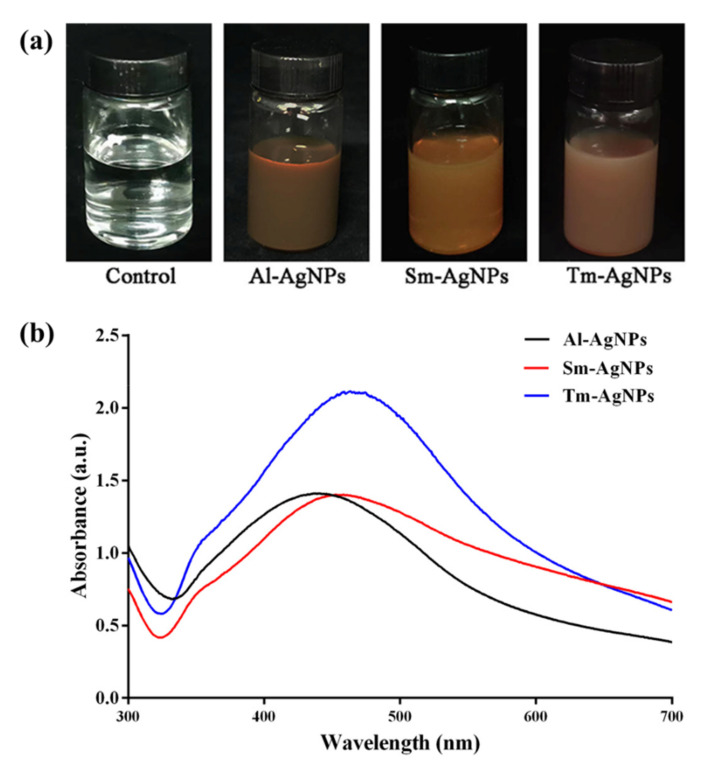
The synthesis of Al-AgNPs, Sm-AgNPs and Tm-AgNPs. (**a**) The color changes in the solutions. (**b**) UV-Visible spectroscopy.

Compared with the control, the colorless solution changed to various browns after reaction with different plant extracts (Figure 1a), indicating the formation of AgNPs. This phenomenon was similar to that described by Melkamu et al. [40], who synthesized AgNPs by the *Hagenia abyssinica* (Bruce) J.F. Gmel. The color intensity of the solution depends on the different plant extracts. The brown color in the AgNPs colloidal solution results from surface plasmon vibrations excitation [41].

UV-Visible spectroscopy is a powerful technology for detecting the existence of nanoparticles. Generally, AgNPs possess absorption peaks at 400–500 nm [42]. Results from this study revealed the UV-Visible spectroscopy of AgNPs mediated by the three plants with absorption peaks at 438 nm, 458 nm, and 463 nm, respectively (Figure 1b). These absorption peaks were similar to the synthesized AgNPs mediated by *Phyllanthus emblica* fruit and *Cardamom* fruit [43,44].

Thus, based on this study’s color changes and absorption peaks, we concluded that three AgNPs were successfully biosynthesized using *A. lappa* fruit, *S. melongena* leaves and *T. mongolicum* leaves, which were designated as Al-AgNPs, Sm-AgNPs, and Tm-AgNPs, respectively.

### 2.2. Characterization of AgNPs

**Figure 2:**
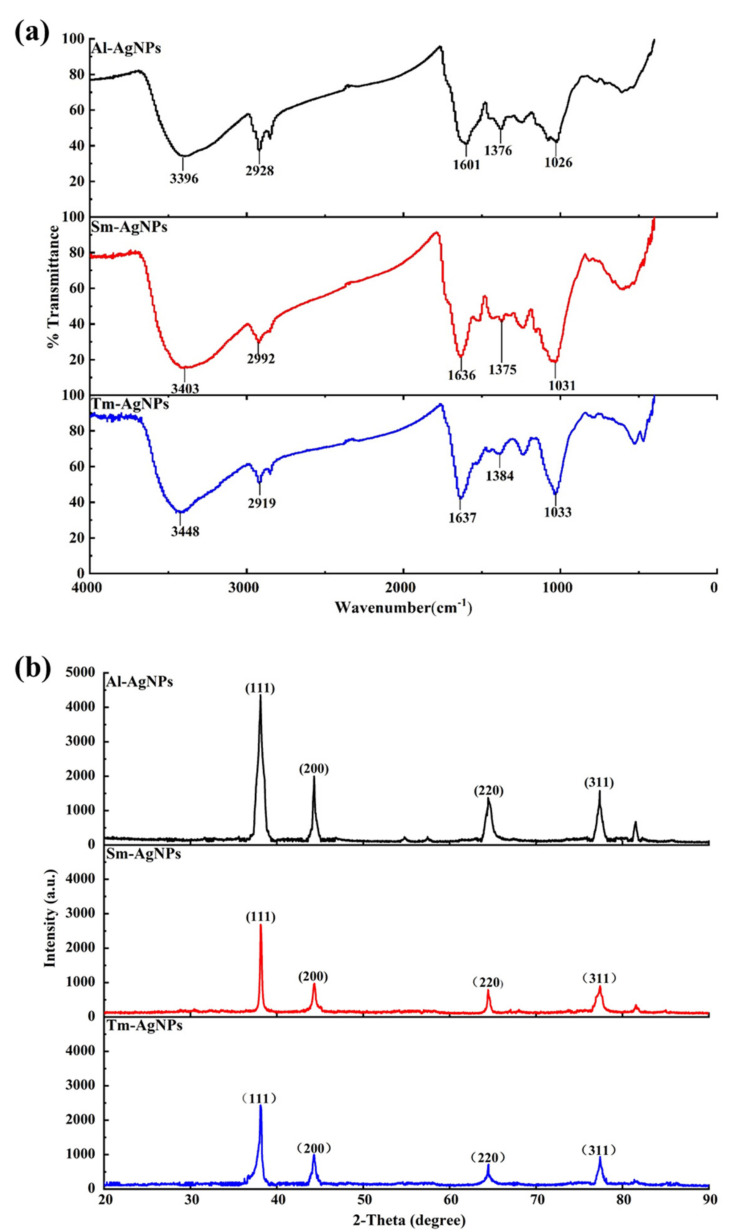
Characterization analysis of Al-AgNPs, Sm-AgNPs and Tm-AgNPs. (**a**) FTIR analysis (**b**) XRD analysis.

FTIR analysis revealed the functional groups of biological materials that coated on AgNPs (Figure 2a). The bands of AgNPs synthesized by three plants appeared at 3396, 3403, and 3448 cm^−1^, attributed to O-H stretching, demonstrating the presence of phenol and alcohol in plant aqueous solutions. The peaks figured at about 2928, 2992, and 2919 cm^−1^ attributed to C-H stretching. The bands at 1601, 1636, and 1637 cm^−1^ were designated as C=O of amide groups, while the bands at 1376, 1375, and 1384 cm^−1^ represented the C-N of aromatic amines. Moreover, owing to the C-O stretching of carboxylic acid, the spectra peaks appeared at 1026, 1031, and 1033 cm^−1^. The FTIR results showed terpenoids, phenolic compounds, and flavonoids could be used as capping agents and stabilizers to synthesize AgNPs. Our study proved the FTIR results of Taghavizadeh et al. [45].

The XRD of AgNPs synthesized by the three plants is shown in Figure 2b. The diffraction peaks of Al-AgNPs appeared at 38.117, 44.294, 64.503, and 77.411, and the Sm-AgNPs showed peaks at 38.137, 44.335, 64.463, and 74.411. In addition, the peaks of the Tm-AgNPs at 38.076, 44.314, 64.463 and 77.370. These peaks correspond to (111), (200), (220), and (311) silver diffraction planes, indicating that three synthesized AgNPs have four different face-centered cubic planes. Three synthesized AgNPs had a single diffraction peak, which proved that the purity and crystallinity of the synthesized AgNPs are good. The spectral intensity of Al-AgNPs was higher, indicating a high level of crystallinity. Our XRD results confirmed the study of Aziz et al. [46], who synthesized AgNPs by *Piriformospora indica*.

**Figure 3:**
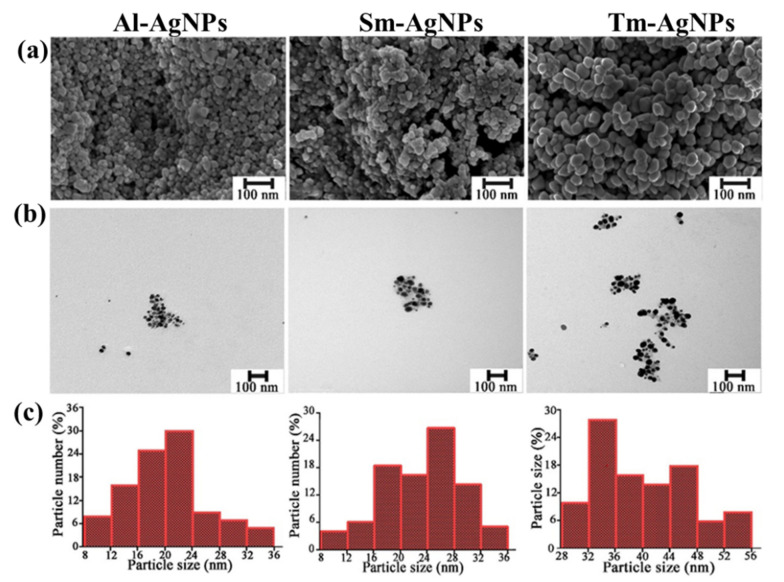
Morphology and size of Al-AgNPs, Sm-AgNPs, and Tm-AgNPs, scale bar = 100 nm. (**a**) SEM image (**b**) TEM image. (**c**) Size distribution of AgNPs.

We studied the morphology, shape, and size of AgNPs through TEM and SEM analysis to better understand the characteristics of AgNPs. The SEM analysis showed that small-sized particles with almost uniform shapes were closely packed together to form a complex structure with a smooth surface (Figure 3a). In addition, several intergranular cavities were also observed (Figure 3a). The size of Al-AgNPs and Sm-AgNPs varied between 8 nm and 36 nm. In addition, the Tm-AgNPs showed a diameter in the range of 28-56 nm (Figure 3c). The average diameters of the three synthesized were 20.18 nm, 21.00 nm, and 40.08 nm, respectively. The TEM analysis showed the three synthesized AgNPs were mostly spherical (Figure 3b), which was similar to the studies of Sharifi-Rad et al. [47]. Moreover, the slight agglomeration of AgNPs was also observed (Figure 3b). Small size nanoparticles have a larger specific surface area, which is more likely to accumulate on cells and subsequently cause cell wall depression, leading to effective cell destruction [48,49].

### 2.3. Antibacterial Activity of AgNPs

**Figure 4:**
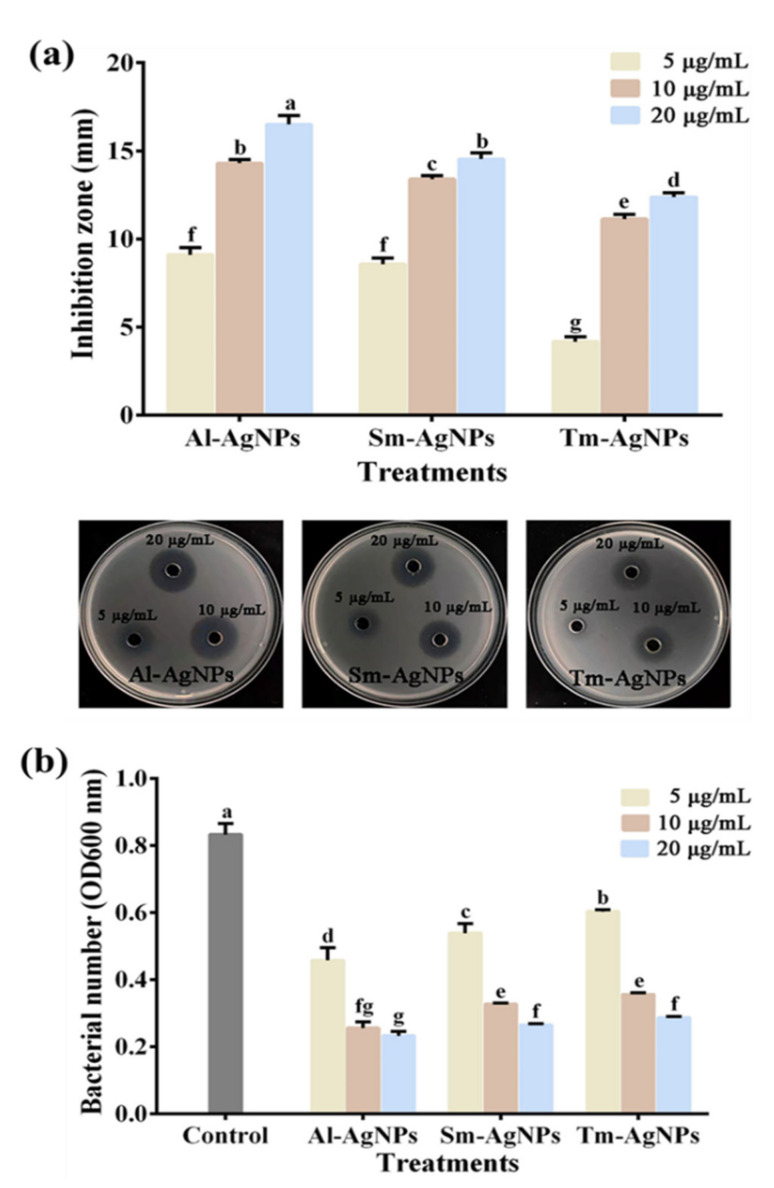
The antibacterial effect of Al-AgNPs, Sm-AgNPs and Tm-AgNPs on Xoo strain C2. (**a**) The inhibition zone of well diffusion assay; (**b**) The in vitro antibacterial activity in liquid broth. Error bars showing the mean of three replicates (n = 3 ± standard deviation). Different lowercase letters on the bars are represent the signifcantly different at *p* ≤ 0.05.

The AgNPs biosynthesized by three plants showed good in vitro antagonistic activity against Xoo strain C2 in Semi-solid agar media (Figure 4a). Indeed, the bacterial growth inhibition zone was clearly observed. When the concentration of three kinds of AgNPs were 5, 10, and 20 μg/mL, the diameters of inhibition zones generated by Al-AgNPs were 9.1 mm, 14.1 mm, and 16.5 mm, respectively, and those generated by Sm-AgNPs were 8.6 mm, 13.4 mm, and 14.5 mm, respectively. The diameters of inhibition zones produced by Tm-AgNPs were 4.2 mm, 11.1 mm, and 12.4 mm, respectively. Similarly, Ulagesan et al. [50] discovered that the inhibition zone produced by AgNPs in the solid culture of *Pseudomonas aeruginosa* is related to the concentration. The higher the concentration, the larger the inhibition zone.

The three biosynthesized AgNPs represented good in vitro antagonistic activity against Xoo strain C2 in liquid broth (Figure 4b). Indeed, 5 μg/mL Al-AgNPs, Sm-AgNPs and Tm-AgNPs decreased the OD_600_ values by 45.08%, 35.33%, and 27.61%, respectively. In addition, Al-AgNPs, Sm-AgNPs and Tm-AgNPs at 10 μg/mL caused the decrease by 69.27%, 60.78% and 57.26% of OD_600_ value, respectively. Furthermore, 20 μg/mL Al-AgNPs, Sm-AgNPs and Tm-AgNPs resulted in the decrease of OD_600_ value by 72.10%, 68.19%, and 65.60% respectively. Ogunyemi et al. [2] observed the antibacterial activity of MgO, MnO_2_ and ZnO nanoparticles against bacterial leaf blight pathogen. As compared to the results of Ogunyemi et al. [2], our study found that the synthesized AgNPs exhibited stronger antibacterial activity in liquid medium.

Similarly, Mishra et al. [51] used 20–50 μg/mL AgNPs synthesized by *Stenotrophomonas* sp. BHU-S7 to inhibit Xoo. Compared with the study of Mishra et al. [51], Al-AgNPs, Sm-AgNPs and Tm-AgNPs can inhibit Xoo at a lower concentration. The antibacterial effect of AgNPs against Xoo strain C2 depend on both its concentration and kind. Generally, the higher the concentration of AgNPs, the stronger the inhibitory effect of the three biosynthetic AgNPs. Among Al-AgNPs, Sm-AgNPs, and Tm-AgNPs, the greatest inhibitory effect was achieved by the Al-AgNPs under the same concentration, which may be due to the size of AgNPs. Our study makes clear that the smaller the nanoparticles, the better the antibacterial effect of AgNPs, which is in accordance with the study of Dong et al. [52].

### 2.4. Antibacterial Mechanism of AgNPs

Many possible antibacterial mechanisms of AgNPs have been reported. Interestingly, our results indicate that the antibacterial activity of the synthesized AgNPs may be mainly attributed to their both direct killing and indirect effects in biofilm formation, swimming, and cell integrity.

#### 2.4.1. Bacterial Biofilm

**Figure 5:**
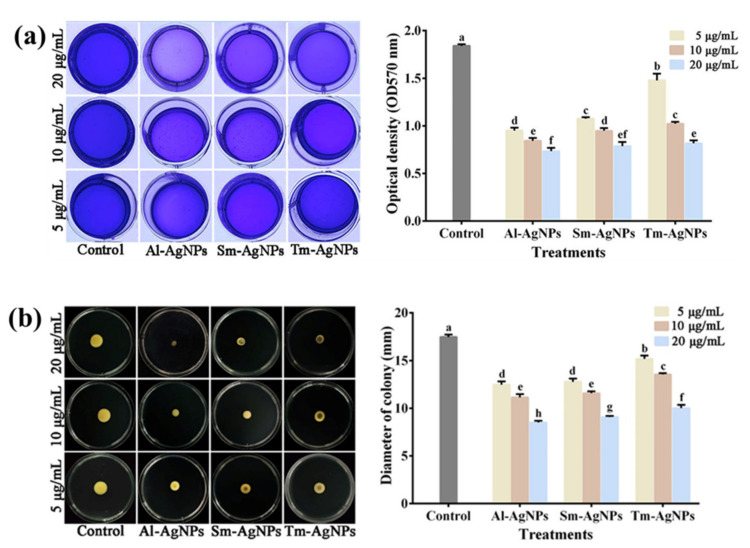
Inhibition of Al-AgNPs, Sm-AgNPs and Tm-AgNPs on biofilm formation and swimming motility of Xoo strain C2. (**a**) The inhibition of biofilm formation. (**b**) The inhibition of swimming motility. Error bars showing the mean of three replicates (n = 3 ± standard deviation). Different lowercase letters on the bars are represent the signifcantly different at *p* ≤ 0.05.

Biofilm formation is essential for the attachment, colonization, virulence, and antibiotic resistance of plant pathogens [53]. The results indicated that the three AgNPs synthesized by different plants strongly inhibited bacterial biofilm formation (Figure 5a). The OD570 values represents the amount of biofilm formation. Indeed, Al-AgNPs, Sm-AgNPs, and Tm-AgNPs at 5 μg/mL led to 48.70%, 41.59%, and 20.53% reduction in the OD570 values of Xoo strain C2, respectively. In addition, Al-AgNPs, Sm-AgNPs, and Tm-AgNPs at 10 μg/mL decreased the OD570 values of Xoo strain C2 by 55.08%, 48.53%, and 45.00%, respectively, while 20 μg/mL Al-AgNPs, Sm-AgNPs, and Tm-AgNPs attenuated biofilm formation at 60.41%, 57.3%, and 56.14%, respectively. In agreement with the antibacterial effect, the reduction of biofilm formation is related to the kind and concentration of AgNPs. Thus, we conclude that the antibacterial activity of three biosynthesized AgNPs may be partially due to their inhibition in biofilm formation. Similarly, Hossain et al. [54] found that AgNPs could reduce biofilm formation of *Pseudomonas rhodesiae*.

#### 2.4.2. Swimming Inhibition

Previous studies showed that bacterial swimming is directly related to bacterial growth, biofilm formation, and pathogenesis [55,56]. In this study, the AgNPs synthesized by three plants showed a strong inhibitory effect on bacterial swimming (Figure 5b). Indeed, Al-AgNPs, Sm-AgNPs, and Tm-AgNPs at 5 μg/mL decreased the swimming ability of Xoo strain C2 by 28.76%, 26.9% and 13.74%. In addition, 10 μg/mL Al-AgNPs, Sm-AgNPs and Tm-AgNPs attenuated the swimming ability of Xoo strain C2 by 36.40%, 33.77%, and 22.52%. Al-AgNPs, Sm-AgNPs, and Tm-AgNPs at 20 μg/mL weakened the swimming ability of Xoo strain C2 by 51.43%, 48.08% and 42.75%. Consistent with the antibacterial effect, the weakening of bacterial swimming ability is related to the kind and concentration of AgNPs. Thus, we speculate that the three AgNPs can better achieve their antibacterial effects by inhibiting the swimming motility of bacteria. Our study is similar to Saeki et al. [55], who reported that AgNPs can weaken the swimming diameter of *Pseudomonas aeruginosa.*

#### 2.4.3. Direct Killing Assay

Previous studies demonstrated that the biogenic AgNPs can change the permeability of cell membrane, enter cells, and kill cells [57,58]. The inhibition effect of AgNPs on bacteria was explored based on the observation of the bacterial staining [59,60].

**Figure 6:**
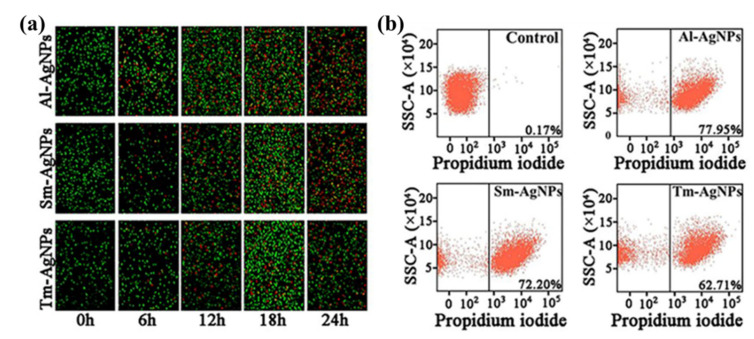
Direct killing effect of Al-AgNPs, Sm-AgNPs and Tm-AgNPs on Xoo strain C2 (**a**) Live/dead cell staining analysis. (**b**) Flow cytometry analysis.

Propidium iodide (PI) and SYTO 9 are fluorescent dyes in the live/dead bacteria staining kit with two different colors. PI can dye dead bacteria with damaged cell membrane red, and SYTO 9 can dye live bacteria with intact cell membrane green. In this study, the AgNPs synthesized by three plants showed direct killing effect on Xoo strain C2. We used the live/dead staining kit to stain the bacteria treated and untreated with AgNPs. In the absence of AgNPs, almost all bacteria on the image had green fluorescence (Figure 6a). Under the same treatment time, the red fluorescence intensity of bacteria treated with Al-AgNPs was higher than that of Sm-AgNPs and Tm-AgNPs. In addition, the results showed that the red fluorescence of the three AgNPs treated bacteria was the strongest at 24 h (Figure 6a). The study by Ahmed et al. [61] showed that after being treated with ZnONPs, *Burkholderia glumae*, and *Burkholderia gladioli* were stained red by the live/dead bacteria staining was similar to our finding.

PI staining combined with flow cytometry can be used to quantitatively record the bacterial mortality after AgNPs treatment [62,63]. We treated Xoo strain C2 with three AgNPs to perform quantitative analysis of bacterial death by flow cytometry. Flow cytometry assay indicated that Al-AgNPs caused the death of 77.95% of Xoo strain C2, Sm-AgNPs caused the death of 72.20% of Xoo strain C2 and Tm-AgNPs caused 62.71% death of Xoo strain C2 (Figure 6b). However, the death rate of Xoo strain C2 without AgNPs treatment was only 0.17% ((Figure 6b). Our results show that the three synthesized AgNPs can cause a large number of bacterial deaths. The study is similar to Cheng et al. [64], who recorded the number of *Ralstonia solanacearum* killed by the synthesized AgNPs by flow cytometry and PI staining.

Living and dead bacteria staining analysis showed that Xoo strain C2 was almost all alive without AgNPs. Regardless of the type of AgNP, bacterial mortality was positively correlated with time. Under the same time treatment, the bacterial mortality of Al-AgNPs treatment was higher than that of Sm-AgNPs and Tm-AgNPs treatment. In agreement with the results of antibacterial effect, greater mortality of Xoo strain C2 was achieved by the Al-AgNPs in both PI staining and Flow cytometry assay.

#### 2.4.4. Ultrastructural Observation of Bacteria

**Figure 7:**
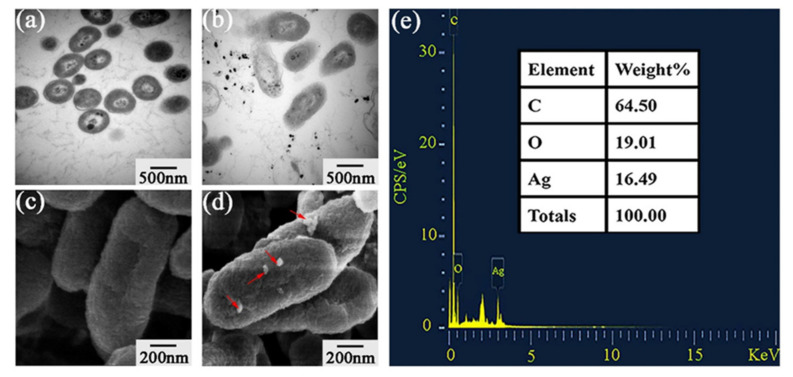
Ultrastructural observation of Xoo strain C2 after 24 h treatment with/without 20 µg/mL Al-AgNPs. (**a**) TEM images of bacterial without Al-AgNPs. (**b**) TEM images of bacterial with Al-AgNPs. (**c**) SEM images of bacterial without Al-AgNPs. (**d**) SEM images of bacterial with Al-AgNPs. (**e**) EDS analysis of bacteria treated with Al-AgNPs.

Due to Al-AgNPs has the strongest inhibitory effect, we used Al-AgNPs for further study. The TEM observation in the study revealed the ultrastructural changes in Xoo strain C2 by Al-AgNPs. Indeed, the bacteria morphology was complete, and the cell content was sufficient in the absence of Al-AgNPs (Figure 7a). In contrast, the morphology of bacteria treated with Al-AgNPs was seriously damaged and appeared deformation and rupture. The surface of the bacteria was shrunk and twisted, and a large number of cytoplasm leaked (Figure 7b). As a kind of Gram-negative bacteria with a negative charge, Xoo has electrostatic attraction to AgNPs with positive charge. Compared with the bacteria without Al-AgNPs treatment (Figure 7c), the surface of the fine army treated with Al-AgNPs had white patches (Figure 7d). The results of SEM showed that the Al-AgNPs could be adsorbed on the surface of Xoo strain C2.

The EDS analysis of bacteria proved that there was Ag element on the surface of Xoo strain C2 treated with Al-AgNPs, which further verified the scanning electron microscope observation results (Figure 7e). In agreement with our result, Ansari et al. [65] showed that AgNPs be adsorbed on bacteria cell wall and penetrate into the bacteria, bringing about changes in the bacterial structure and ultimately leading to bacterial death. Due to the attachment of silver ions to negatively charged cell surfaces, AgNPs can directly alter cell processes, including permeability, electron transport and osmoregulation, which can lead to the release of bacterial DNA [28,66].

## 3. Materials and Methods

### 3.1. Plant Materials

*A. lappa* fruit, *S. melongena* leaves, and *T. mongolicum* leaves were collected from Hangzhou, Zhejiang Province, China. Xoo strain C2 was cultured in nutrient agar (NA) at 30 °C for 24 h to obtain single bacterial colony. The single colony of Xoo strain C2 was cultured in nutrient broth (NB) at 30 °C and 200 rpm for 24 h to reach the logarithmic growth period of bacteria.

### 3.2. Preparation of the Three Plant Extracts

Plant aqueous extracts were prepared according to the procedure described by instructions of Khatami et al. [67]. In brief, the *A. lappa* fruit, *S. melongena* leaves and *T. mongolicum* leaves were cut and dried. After pulverizing the three dried plant materials into powder with mortar and pestle, 400 mL of double distilled H_2_O (ddH_2_O) was added to three plant powder (4 g). After stirring, three 1% (*w*/*v*) plant water mixtures were obtained. Then, the resulting mixtures were centrifuged to obtain three supernatants.

### 3.3. Green Synthesis of AgNPs

The synthesis method for AgNPs was modified based on the method of Miri et al. [68]. In detail, 100 mL of 2 mM AgNO_3_ solution were mixed with 60 mL three different plant extracts respectively in conical flasks and the three mixtures were shaken at 30 °C and 200 rpm for 3 h under shading. When the three solutions turned dark brown, the y were centrifuged at 13,000× *g* for 10 min. Furthermore, washing with ethanol was performed three times by suspending and palleting the 1 g of three AgNPs in deionized H_2_O. Then, the three precipitates were washed with deionized H_2_O. In addition, the Alpha 1–2 LD plus (Christ, Osterode, Germany) was used to freeze dry the three AgNPs powders.

### 3.4. Characterization of AgNPs

The three AgNPs solution at 200 μg/mL were prepared by dissolving the collected three AgNPs powder in ddH_2_O. The successful synthesis of three AgNPs was detected by ultraviolet visible spectroscopy. Using a UV-Visible spectrophotometer (Shimadzu, Kyoto, Japan) the absorption peak of the solution was measured at 300–700 nm according to the method proposed by Masum et al. [43]. The functional groups of biosynthesized AgNPs can be analyzed by the Fourier transform infrared spectroscopy (FTIR, Vector22, Bruker, Bremen, Germany) at 4000–450 cm^−1^ according to Reddy et al. [69]. The crystalline properties of three dried AgNPs were analyzed by xpert Pro diffractometer (XRD) (Siemens D5000, Garching, Germany) as described by Aziz et al. [46], while the measurement voltage of the diffractometer is 40 KV, the current is 30 Ma, the recording range of 2θ is 20–90° and the scanning speed is 6°/min. The morphology of three AgNPs was analyzed using scanning electron microscope (SEM) (SU8010, Hitachi, Tokyo, Japan) and transmission electron microscopy (TEM) (JEM 1230-JEOL, Akishima, Japan). Image J software (v1.8.0, National Institutes of Health) was used to measure the diameter of the synthesized AgNPs.

### 3.5. Antibacterial Activity of AgNPs

#### 3.5.1. Well Diffusion Assay

A well diffusion assay was used to determine the antibacterial activity of AgNPs against Xoo strain C2, based on the method of Masood et al. [70]. In brief, bacterial plates were prepared by mixing the 1 mL overnight bacterial culture (1 × 10^8^ CFU/mL) with 9 mL NA semi-solid medium. We used a sterilized 7-mm perforator to punch holes in the bacterial plate. The Al-AgNPs, Sm-AgNPs and Tm-AgNPs colloids were added to the different bacterial plates respectively. The hole of each bacterial culture plate contained concentrations of 5, 10, and 20 μg/mL of the same type of AgNPs colloids. Then, the bacterial plates were stably placed at 30 °C for 24 h. We measured the diameter of the inhibition zone formed around the hole (minus the diameter of the puncher) to evaluate the ability of three AgNPs to inhibit Xoo strain C2. This experiment was independently repeated three times.

#### 3.5.2. Minimum Inhibitory Concentration Assay

We explored the inhibition of Xoo strain C2 by three colloids of AgNPs in liquid [71]. In brief, 100 μL of three AgNPs colloids (10, 20, 40 μg/mL) were mixed with 100 μL Xoo strain C2 bacterial solution (OD_600_ = 0.4), respectively, in the 96-well plate (Corning, NY, USA). The final concentrations of the three AgNPs colloids in the mixture are 5, 10, and 20 μg/mL. Then, the 96-well plate was cultivated without shaking at 30 °C for 24 h. The control was a mixture of 100 μL sterile water and 100 μL Xoo strain C2. We used a microplate (Thermo Fisher Scientific Inc., Waltham, MA, USA) to measure the density of all samples at OD_600_. The experiment was repeated 3 times independently, and three repeats were set for each to ensure the experimental results’ reliability.

#### 3.5.3. Biofilm Formation Inhibition Assay

Based on Hossain et al. [54], the method of AgNPs inhibiting biofilm formation of Xoo strain C2 was improved. In brief, 100 μL AgNPs colloids (10, 20, 40 μg/mL) were added to100 μL Xoo strain C2 bacterial solution (OD_600_ = 0.4) to obtain the mixture with AgNPs concentration of 5, 10 and 20 μg/mL, respectively. The 200 μL mixture was added to a 96-well plate (Corning, NY, USA), stably cultured at 30 °C for 48 h. The negative control is the bacterial culture with an equal volume of sterile water. Subsequently, the liquid was poured out, and the biofilm was gently washed three times with ddH_2_O. Finally, 100 μL 1% (*w*/*v*) crystal violet aqueous solution was added to dye the-dried biofilm for 30 min, and then add 100 μL 33% acetic acid to dissolve the dye. The intensity of the sample at OD570 nm was measuring using a microplate (Thermo Fisher Scientific Inc., Waltham, MA, USA). This experiment was independently repeated three times.

#### 3.5.4. Swimming Motility Assay

The method for inhibiting AgNPs on bacterial swimming was modified based on Ibrahim et al. [72]. Bacterial droplets were inoculated into the center of NA medium (0.3%, *w*/*v*) containing AgNPs with the concentrations of 0, 5, 10, and 20 μg/mL. Following the incubation at 30 °C for 48 h, the diameter of the bacterial colony was measured to evaluate the swimming ability of Xoo strain C2. This experiment was independently repeated three times.

#### 3.5.5. Live/Dead Cell and Flow Cytometry Assay

The live/dead bacterial cells were detected using the BacLight bacterial viability Kit (Thermo Fisher Scientific, Waltham, MA, USA), which contains SYTO 9 dye (SYTO 9) and propidium iodide dye (PI). This study slightly modified the experimental method of Cheng et al. [64]. In brief, the overnight cultures of Xoo strain C2 were centrifuged (5000× *g*, 6 min) to acquire bacterial pellets, suspended in 20 µg/mL Al-AgNPs, Sm-AgNPs and Tm-AgNPs, respectively, and then incubated in a shaker at 30 °C and 180 rpm for different times. In the dark, all bacterial samples were stained with SYTO 9 and PI for 20 min. Fluorescence was detected using the Olympus inverted confocal microscope (Leica-SP8, Heidelberg, Germany), according to the method of Chandrasekharan et al. [73] with slight adjustments. The overnight bacteria were suspended in the 20 μg/mL concentration of three synthetic AgNPs, then cultured in a shaker at 30 °C, 180 rpm for 24 h. Bacteria resuspended in 0.1 M PBS were used as controls. Following the staining with PI dye for 20 min in the shade, the PI staining bacterial cells were detected by the FACSVerse cytometer (BD Biosciences, San Jose, CA, USA).

#### 3.5.6. Electron Microscopy Observation

The morphological changes in bacterial pathogen at the cellular level were observed after exposure to AgNPs using electron microscopy. Xoo strain C2 was incubated in NB broth with Al-AgNPs at 20 μg/mL at 30 °C, 180 rpm. The obtained bacterial culture was centrifuged at 5000× *g* for 5 min to obtain bacterial pellet, which was washed with PBS. The 0.5 g pellet was resuspended in 0.1M PBS containing 2.5% (*v*/*v*) glutaraldehyde and stored overnight at 4 °C. The bacterial sample was treated with 1% (*w*/*v*) osmic acid fixative for 30 min, and the excess fixative was rinsed with PBS. After that, the sample was fixed in different concentration gradient ethanol solutions (50–100%, *v*/*v*) for dehydration. The treated sample was infiltrated and embedded, then using the TEM (JEM-1230, JEOL, Akishima, Japan) to observe the bacterial structure. After gold spraying, the surface morphology of the treated sample can be observed under SEM (SU8010, Hitachi, Tokyo, Japan). Moreover, Energy dispersive X-ray spectrometry (EDS; Hitachi, Tokyo, Japan) was used to check the elements on the bacterial surface.

## 4. Conclusions

In summary, the three AgNPs were biosynthesized in this study using *A. lappa* fruit, *S. melongena* leaves, and *T. mongolicum* leaves, which were confirmed based on the color change and absorption peak. FTIR, XRD, TEM, and SEM were used to better characterize the three synthesized AgNPs. The biogenic AgNPs were provided with excellent antibacterial activity against Xoo strain C2 in both solid and liquid medium. The antibacterial mechanism may be attributed to the direct killing effect and the indirect effect in biofilm formation, swimming, and cell integrity. Overall, AgNPs synthesized from three plant extracts can well inhibit Xoo strain C2, and Al-AgNPs has the strongest inhibitory effect. This study indicates that the three AgNPs, in particular Al-AgNPs, possess the great latent capacity to the control rice bacterial leaf blight.

## Data Availability

All data of conclusions are included in this paper.
